# Nuclear receptors and metabolism: from feast to famine

**DOI:** 10.1007/s00125-014-3209-9

**Published:** 2014-03-12

**Authors:** Suk-Hyun Hong, Maryam Ahmadian, Ruth T. Yu, Annette R. Atkins, Michael Downes, Ronald M. Evans

**Affiliations:** 1 Gene Expression Laboratory, Salk Institute for Biological Studies, La Jolla, CA 92037 USA; 2 Howard Hughes Medical Institute, Salk Institute for Biological Studies, La Jolla, CA USA

**Keywords:** Circadian metabolism, FGFs, Glucose homeostasis, Nuclear receptors, Review

## Abstract

The ability to adapt to cycles of feast and famine is critical for survival. Communication between multiple metabolic organs must be integrated to properly metabolise nutrients. By controlling networks of genes in major metabolic organs, nuclear hormone receptors (NHRs) play central roles in regulating metabolism in a tissue-specific manner. NHRs also establish daily rhythmicity by controlling the expression of core clock genes both centrally and peripherally. Recent findings show that many of the metabolic effects of NHRs are mediated through certain members of the fibroblast growth factor (FGF) family. This review focuses on the roles of NHRs in critical metabolic organs, including adipose tissue, liver and muscle, during the fed and fasted states, as well as their roles in circadian metabolism and downstream regulation of FGFs.

## Introduction

The ability to navigate through unpredictable environmental changes such as cycles of feast and famine is critical for the survival of an organism. In order to adapt to fluctuations in nutrient availability, communication between multiple metabolic organs must be integrated to properly metabolise carbohydrates, proteins and lipids. Dysregulation of these processes can have an impact on survival during a fast or lead to obesity and related pathologies, such as type 2 diabetes, in response to overnutrition. However, the underlying mechanisms that maintain metabolic balance during extremes of nutrient challenge remain poorly understood.

Insight into these mechanisms can be gained by studying the highly conserved nuclear hormone receptor (NHR) superfamily. The 48 human NHRs are genetic switches that control networks of genes in response to various physiological cues and orchestrate a diverse range of biological functions, including nutrient homeostasis [1–3]. Typical nuclear receptors are comprised of distinct functional domains, including an N-terminal transactivation domain, a highly conserved DNA-binding domain (DBD) and a C-terminal ligand-binding domain containing a ligand-dependent transactivation function [1, 2]. Ligand binding induces a conformational change in the receptor that allows for differential recruitment of cofactors and subsequent modulation of NHR activity [3–6]. A unique feature of many of these receptors is their ability to be activated by lipophilic ligands derived from dietary nutrients such as long chain fatty acids and sterols.

By controlling networks of genes in key metabolic organs, NHRs play critical roles in regulating the metabolism of nutrients in a tissue-specific manner [7]. NHRs also regulate the expression of core clock genes both centrally and peripherally to coordinate and establish daily rhythmicity. Recent studies show that certain members of the fibroblast growth factor (FGF) family are direct targets of NHRs and responsible for mediating many of their metabolic effects. Their importance in metabolism, combined with the ability to manipulate their ligand-dependent activity, make NHRs attractive drug targets for the treatment of metabolic disease. Here, we discuss the roles of NHRs, during feast and famine, in adipose tissue, liver and muscle, as well as their roles in establishing circadian rhythms and regulating FGFs.

## Adipose tissue

White adipose tissue (WAT) is the major energy reserve in mammals. The primary function of WAT is to synthesise and store triacylglycerol during periods of energy excess and to hydrolyse triacylglycerol to generate fatty acids and glycerol for use by other organs during periods of energy deprivation [8]. WAT also secretes adipokines such as leptin and adiponectin that regulate energy intake, metabolism and insulin sensitivity. Excess storage of triacylglycerol in WAT results in obesity and related disorders, including type 2 diabetes. Paradoxically, the metabolic abnormalities found in obesity are also found in lipodystrophies, which are characterised by the inability to properly store fat in adipose tissue. Therefore, an appropriate capacity to store triacylglycerol in WAT in response to changes in nutrient availability is critical for metabolic homeostasis.

**Fig. 1:**
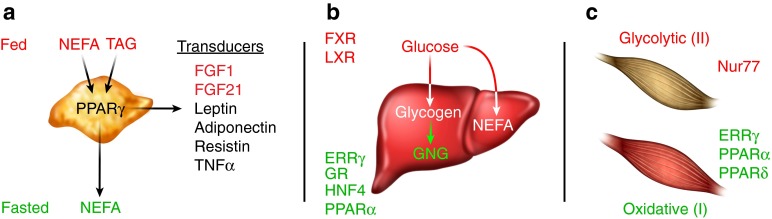
(**a**) Adipose tissue during feast and famine. During the fed state, white adipose tissue (WAT) synthesises and stores energy in the form of triacylglycerol (TAG). During the fasted state, WAT hydrolyses TAG to generate NEFA that can be taken up by other organs and used for energy production. Peroxisome proliferator-activated receptor (PPAR)γ plays a major role in controlling the expression of genes involved in adipogenesis and lipogenesis. PPARγ also controls the expression of secreted factors (transducers) involved in glucose and energy homeostasis such as leptin, adiponectin, resistin and TNFα as well as fibroblast growth factor (FGF)1 and FGF21, which act locally in adipose tissue during the fed state to promote adipose remodelling and differentiation, respectively. (**b**) Liver during feast and famine. During the fed state, excess glucose is taken up by the liver and stored as glycogen via glycogenesis or converted to NEFA through de novo lipogenesis for synthesis and storage as TAG. During the fasted state, glycogen is broken down to generate glucose via glycogenolysis. Prolonged fasting leads to de novo glucose synthesis in the liver through gluconeogenesis (GNG). During starvation, when glycogen stores are depleted, the liver uses acetyl-CoA to produce ketones by a process called ketogenesis, where PPARα plays an important role. The glucocorticoid receptor (GR), oestrogen-related receptor γ (ERRγ) and hepatocyte nuclear factor 4 (HNF4) promote gluconeogenesis, while liver X receptor (LXR) and farnesoid X receptor (FXR) suppress gluconeogenesis. During the fed state, high cholesterol levels activate LXR to promote the production of bile acids. High bile acid levels are then sensed by FXR, which inhibits their synthesis to prevent an accumulation of bile acids in the liver. (**c**) Skeletal muscle during feast and famine. Type I fibres preferentially oxidise fatty acids, while type II fibres preferentially metabolise glucose. PPARγ and ERRγ promote a type I fibre type in skeletal muscle and PPARα promotes fatty acid utilisation, while Nur77 promotes glucose utilisation

While multiple NHRs have been shown to affect WAT metabolism, peroxisome proliferator-activated receptor (PPAR) γ is the most critical for proper WAT function [9, 10]. PPARγ is most highly expressed in adipose tissue and is best known for its role in regulating adipogenic and lipogenic pathways. Generation of whole-body and adipose-specific PPARγ knockout mice revealed that PPARγ is not only required for adipocyte differentiation but also for mature adipocyte function [11–16]. PPARγ also plays a critical role in glucose homeostasis by increasing the expression of GLUT4 and c-Cbl-associated protein, as well as numerous secreted factors that affect insulin sensitivity, such as adiponectin, resistin, leptin and TNFα in WAT [17–20] (Fig. 1a). In mice, activation of PPARγ specifically in adipocytes is sufficient to cause whole-body insulin sensitisation and, conversely, a dominant-negative mutation in a single allele of *PPARG* in humans leads to partial lipodystrophy and insulin resistance, supporting its role in adipogenesis and insulin sensitivity [21–24]. The thiazolidinediones (TZDs), which are known agonists of PPARγ, exhibit potent adipogenic and glucose-lowering effects, and, despite their unwanted side effects, remain highly effective for the treatment of metabolic disease [25].

## Liver

Maintenance of blood glucose level is a primary requirement for survival. Although multiple organs are involved in glucose homeostasis, the liver is the major player. During the fed state, excess glucose is taken up by the liver and stored as glycogen via glycogenesis or converted into fatty acids (de novo lipogenesis) for synthesis/storage as triacylglycerol. During the fasted state, stored glycogen is broken down by the liver to generate glucose by a process called glycogenolysis. Prolonged fasting or starvation induces de novo glucose synthesis in the liver through hepatic gluconeogenesis. During starvation, when glycogen stores are depleted and there is a reliance on fatty acids, the liver also uses acetyl-CoA to produce ketones, by a process termed ketogenesis.

Several NHRs have been implicated in the control of glucose homeostasis in the liver (Fig. 1b). The first NHR to be linked with hepatic gluconeogenesis was the glucocorticoid receptor, which is activated by cortisol. During fasting, increased secretion of cortisol from the adrenal gland increases the activity of the glucocorticoid receptor in the liver, leading to transcriptional activation of hepatic gluconeogenic genes such as glucose-6-phosphatase (*G6PC*) and phosphoenolpyruvate carboxykinase (*PEPCK*, also known as *PCK1*). Other NHRs, such as hepatocyte nuclear factor 4 (HNF4) and oestrogen-related receptor γ (ERRγ) have also been shown to promote hepatic gluconeogenesis during fasting [26, 27]. PPARα is also critical in the liver during the adaptive response to fasting. PPARα null mice do not present any overt phenotype under standard animal housing conditions. However, when fasted, they exhibit severe hypoglycaemia and impaired ketogenesis [28–30]. Interestingly, the fatty acid synthase-dependent phospholipid 1-palmitoyl-2-oleoyl-*sn*-glycerol-3-phosphocholine (16:0/18:1-GPC) has been shown to be an endogenous ligand for PPARα [31]. During the fed state, liver X receptor (LXR) α and LXRβ, which are activated by oxysterols, have been shown to suppress gluconeogenic enzymes and upregulate the expression of glucokinase to promote hepatic glucose utilisation [32], while the bile acid responsive farnesoid X receptor (FXR) suppresses gluconeogenic genes and increases glycogen synthesis [33].

In addition to promoting glucose uptake and storage, the liver plays an important role in cholesterol, bile acid, fatty acid and triacylglycerol homeostasis. When cholesterol levels are high, oxysterols generated through cholesterol degradation activate LXR, which in the liver leads to the formation of bile acids, to avoid excess accumulation of cholesterol. High levels of bile acids are sensed by FXR, which inhibits their synthesis in order to prevent an accumulation of bile acids in the liver. During the fed state, LXR activation also leads to increased triacylglycerol accumulation in the liver by increasing the transcription of genes involved in hepatic de novo lipogenesis [34]. While PPARα has also been shown to increase the expression of some genes involved in lipogenesis, it also promotes the uptake and oxidation of fatty acids in mitochondria, peroxisomes and microsomes [35]. PPARα activation leads to lower serum triacylglycerol levels through induction of lipoprotein lipase (LPL) activity via inhibition of apoCIII (LPL inhibitor) expression [35]. In addition to the hypolipidaemic fibrate drugs that target PPARα, it is possible that targeting of other NHRs in the liver may lead to promising therapeutics for the metabolic syndrome.

## Muscle

Skeletal muscle is composed of multiple myofibres that differ in their metabolic properties, including oxidative slow-twitch (type I), mixed oxidative-glycolytic fast-twitch (type IIa) and glycolytic fast-twitch (type IIb) myofibres [36, 37]. Type I fibres preferentially oxidise fatty acids, while type II fibres preferentially metabolise glucose. Adaptation to fasting in skeletal muscle is aimed at sparing glucose by switching to fatty acid oxidation. Suppression of glucose utilisation is accomplished via activation of pyruvate dehydrogenase kinase 4 (PDK4), which phosphorylates and thus inactivates the pyruvate dehydrogenase complex to prevent pyruvate oxidation and conserve lactate and alanine for gluconeogenesis [38]. During the fed state, when insulin levels are high, skeletal muscle handles the majority of glucose disposal in the body.

NHRs play a critical role in controlling glucose and fatty acid metabolism in skeletal muscle (Fig. 1c). Growth factor-inducible immediate early gene nur/77-like receptor (Nur77), a NHR preferentially expressed in glycolytic vs oxidative muscle, promotes the expression of genes involved in glucose metabolism in skeletal muscle [39]. On the other hand, PPARα, which activates PDK4, promotes lipid utilisation and inhibits glucose uptake in skeletal muscle [38, 40]. Mice overexpressing PPARα in skeletal muscle exhibit glucose intolerance but are protected from high fat diet (HFD)-induced obesity, while PPARα null mice exhibit enhanced glucose tolerance, despite increased triacylglycerol accumulation in muscle and HFD-induced obesity [40]. PPARδ also promotes fatty acid utilisation in muscle but, in contrast to PPARα, activation of PPARδ leads to enhanced glucose uptake and improved skeletal muscle insulin sensitivity, as evidenced by studies in mice with genetic ablation and ectopic expression of PPARδ [28, 41–44]. PPARδ has also been shown to activate a genetic programme that induces a type I endurance muscle fibre type switch, leading to an enhanced capacity for endurance running and the ability to oxidise both lipids and carbohydrates at higher rates [42]. In this regard, ERRγ, which is expressed at much higher levels in type I vs type II muscle fibres, has been shown to be critical for maintaining the highly vascularised and oxidative capacity of type I muscle fibres, even in the absence of exercise [45]. This ability to manipulate oxidative/glycolytic metabolism in skeletal muscle through NHRs represents a potential therapeutic avenue for treating metabolic disease [46].

## Circadian rhythms

Mammals experience natural sleep/wake and fed/fasted cycles and most physiological processes are regulated in a circadian manner. These rhythms are generated by a master clock located in the suprachiasmatic nucleus (SCN) of the hypothalamus, which synchronises physiology to day/night cycles. While the master clock, which is entrained by light onto the retina, can synchronise clocks in peripheral tissues, feeding/fasting rhythms also serve as strong entrainment factors for metabolic organs.

**Fig. 2:**
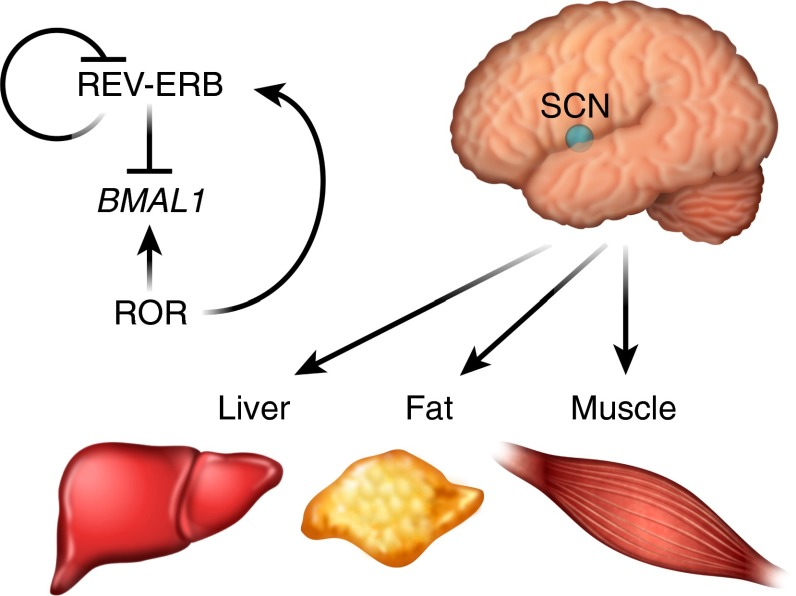
Circadian metabolism. Circadian rhythms are generated by a master clock located in the SCN of the hypothalamus, which is entrained by light on the retina and can synchronise the clocks in peripheral tissues. Feeding/fasting cues also serve as strong entrainment factors for metabolic organs. REV-ERBs and RORs reciprocally regulate the expression of target genes, including *BMAL1*. REV-ERBs act as transcriptional repressors, while RORs act as activators

At the genomic level, NHR signalling is intertwined with the core clock machinery, which consists of the transcription factors circadian locomotor output cycles kaput (CLOCK) and brain and muscle ARNT-like 1 (BMAL1) and their co-repressors period (PER) and cryptochrome (CRY). NHRs can be regulated by the clock and can also regulate the clock themselves. The NHRs reverse c-erbA (REV-ERB)α and REV-ERBβ along with three retinoid orphan receptors (RORα, β, γ) regulate many metabolic pathways in a circadian fashion by binding to target genes with an ROR response element. These receptors reciprocally regulate the expression of target genes, with RORs serving as constitutive activators and REV-ERBs acting as repressors [47] (Fig. 2). The circadian expression pattern of BMAL1 is generated by the oscillating expression of both RORα and REV-ERBα in the SCN [48]. REV-ERBα and REV-ERBβ are also involved in peripheral circadian regulation of liver metabolism by regulating the CLOCK–BMAL1 transcriptomes [49]. Interestingly, haem has been identified as an endogenous ligand for REV-ERBα/β linking metabolism and circadian clock [50, 51]. Furthermore, many genes involved in both clock and metabolic functions harbour BMAL1 and REV-ERBα/β binding sites, indicating an integrated mechanism of circadian gene expression by these transcription factors [49, 52].

The glucocorticoid receptor also plays a critical role in establishing circadian rhythmicity [53]. Circulating levels of glucocorticoids oscillate throughout the day with peak levels during the onset of activity. The glucocorticoid receptor is also critical for resetting the clock in peripheral tissues, such as the liver, and there are glucocorticoid response elements (GREs) in core clock genes such as *PER1* and *PER2*. It has also been shown that CRY directly binds to the glucocorticoid receptor to regulate its activity. In addition to the glucocorticoid receptor, REV-ERBα/β and RORs, many other NHRs have been shown to be expressed in a circadian fashion and play a role in circadian metabolism [54, 55]. In fact, in key metabolic organs such liver, muscle and adipose tissue, over half of the NHRs detected exhibit circadian expression, suggesting that changes in the expression of NHRs and their downstream target genes are likely to explain the cyclical behaviour of glucose and lipid metabolism [55].

## The NHR–FGF connection

**Fig. 3:**
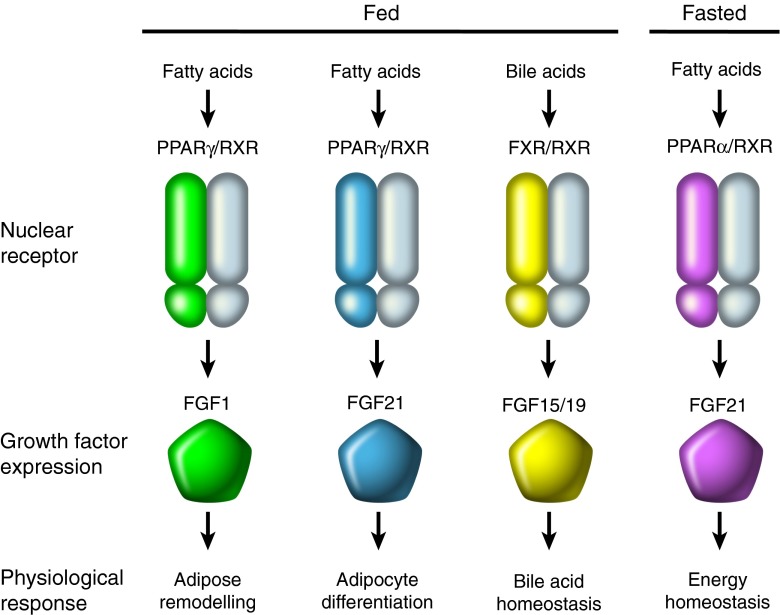
NHR–FGF interface during feast and famine. During the fed state PPARγ, which is activated by fatty acids, increases the expression of both FGF1 and FGF21 to promote adipose remodelling and adipocyte differentiation, respectively. During the fed state, bile acids activate FXR, which increases the expression of FGF15/19 to promote bile acid homeostasis. During the fasted state, fatty acids activate PPARα to control energy homeostasis

Recently, NHRs have been shown to exert control on nutritional homeostasis by regulating the expression of four different members of the FGF family [25, 56]. FGFs are secreted heparan sulphate-binding proteins that act locally by signalling through FGF receptors and are typically involved in cell growth, angiogenesis and wound healing [57, 58]. In contrast, the endocrine FGFs (FGF 19, 21 and 23) have poor affinity for heparan sulphate and therefore circulate rather than being immobilised in the extracellular matrix [58]. While the classical, paracrine FGFs require heparan sulphate for proper activation of FGF receptors, the endocrine FGFs require the α or β Klotho co-receptors for signalling [56, 59]. The endocrine FGFs have received much attention for their role as metabolic regulators and are controlled by NHRs (Fig. 1a). While FGF23, through its interaction with the vitamin D receptor, has been shown to be critical for controlling vitamin D metabolism, we will focus on the FXR–FGF19 and PPAR–FGF21 signalling pathways because of their relevance during feast and famine [56] (Fig. 3).

FGF19 is a postprandial hormone, and the gene encoding this FGF is a direct bile acid-dependent target of FXR. The importance of FGF19 (FGF15 in rodents) in maintaining bile homeostasis has been demonstrated in both rodent studies and human clinical studies. In addition to its importance in bile acid metabolism, FGF19 has also been shown to lower blood glucose, hepatic triacylglycerol and cholesterol levels [60, 61] and induce protein and glycogen synthesis in the liver independently of insulin [62]. However, unlike insulin, FGF19 does not activate the phosphoinositide 3-kinase (PI3K)–Akt signalling pathway but, rather, signals through the Ras–extracellular signal-related kinase (ERK) pathway to promote protein and glycogen synthesis [62, 63]. FGF19 also reduces hepatic gluconeogenesis by repressing the expression of the transcription cofactor PPARγ co-activator 1α (PGC1α), and the PGC1α target genes, *G6PC* and *PEPCK*, also through a mechanism distinct from that of insulin [64]. Unlike insulin, FGF19 does not increase lipogenesis and actually, as mentioned above, reduces hepatic triacylglycerol and cholesterol levels [60, 61]. While there are concerns regarding potential mitogenic effects [65, 66], its overlapping but unique functions with insulin make FGF19 an attractive target for the treatment of both type 1 and 2 diabetes.

In addition to FGF19, FGF21 has emerged as a member of the endocrine FGFs that is regulated by an NHR to control metabolism. Initially identified as a factor induced by PPARα in the liver in response to fasting, FGF21 is now recognised as a key player in the adaptive starvation response, helping to shift hepatic metabolism from carbohydrates to fatty acids and ketone bodies [67, 68]. Interestingly, FGF21 also has an important function in the fed state, where it is induced by PPARγ and works in a paracrine manner in adipose tissue to enhance adipogenesis [68, 69]. FGF21 knockout mice exhibit impaired PPARγ signalling in adipose tissue, reduced adiposity and are resistant to the insulin-sensitising effects, as well as the undesirable side effects, of the TZDs, which, as mentioned previously, are PPARγ agonists [67]. Pharmacological administration of FGF21 reverses hepatic steatosis and improves glucose homeostasis in rodents and non-human primates [70–72]. Recent studies have shown that FGF21 exerts its effects on glucose homeostasis through adiponectin, an adipokine known to enhance insulin sensitivity [73, 74]. It remains to be determined whether other metabolic effects of FGF21 are also mediated through adiponectin. In addition to its effects on peripheral metabolism, FGF21 was recently shown to act centrally to promote adaptations to fasting by increasing glucocorticoid levels, suppressing physical activity and altering circadian behaviour [75]. Prevention of female reproduction is a critical adaptation to starvation, and FGF21 has been shown to exert an inhibitory effect on this process by acting on the SCN in the hypothalamus [76]. Future studies further addressing how the central and peripheral actions of FGF21 are coordinated to control metabolism will be an exciting new avenue of research [75, 76].

While the endocrine family of FGFs has received the most attention for its role in metabolism, a new role for the non-endocrine FGF1 in nutrient homeostasis has emerged. As part of a screen to identify genes that respond to feast and famine cues, FGF1 was found to be induced in WAT in response to HFD and repressed during a fast, pointing to an unexpected metabolic function in both fed-state and fasted-state responses. FGF1 was also shown to be induced in visceral adipose tissue in response to HFD or TZD treatment [77]. Despite its implication in multiple physiological processes, FGF1 knockout mice exhibit no phenotype under standard laboratory conditions, which led to the long-held assumption that it was dispensable [58]. However, when placed on an HFD, FGF1 knockout mice develop marked fibrosis in adipose tissue, structurally restricting adipose tissue expansion and resulting in a severe diabetic phenotype. Even more striking, upon withdrawal of the HFD the adipose tissue in these mice fails to properly contract, leading to severe necrosis of the adipose tissue [77]. The induction of FGF1 expression in adipose tissue in the fed state is regulated at the genomic level by PPARγ, identifying a PPARγ–FGF1 signalling axis crucial for handling cycles of feast and famine.

## Concluding remarks

As global regulators of metabolism, NHRs play critical roles in key metabolic organs during feast and famine. Their importance in metabolism, combined with their ability to be modulated by small lipophilic ligands, make NHRs one of the most highly therapeutically targeted protein families, serving as targets for drugs including glucocorticoids, thyroid hormone, tamoxifen, fibrates and TZDs. Although these NHR-targeted drugs are highly effective, there is the potential to improve on some of these therapies. For example, while highly effective for treating type 2 diabetes, TZDs are accompanied by unwanted side effects, including fluid retention, weight gain, bone loss and congestive heart failure. New strategies are being developed for improving TZD-based therapies, one of which involves targeting downstream effectors of PPARγ, such as FGF21 or, potentially, FGF1. In this regard, it will be important to dissect the relative roles of these two FGFs in adipose tissue, since they are both regulated in the fed state by PPARγ. Recently, a FGF21 analogue has been shown to improve dyslipidaemia in obese people, however only a trend toward glucose lowering was observed [78, 79]. Also, bone loss in these individuals was not examined, which will be important since FGF21 causes bone loss in rodents [80]. While drugs are being developed for FGF21, it will be interesting to see what effect, if any, FGF1 has systemically. Also, the insulin-like properties of FGF19, combined with its ability to lower triacylglycerol and cholesterol levels and its nonlipogenic properties, make it an attractive therapeutic target of the treatment of both type 1 and 2 diabetes. While there are concerns about potential mitogenic effects of FGF19 [65, 66], therapies designed to dissociate its mitogenic effects from its metabolic ones might be possible [81]. Additionally, targeting NHRs that affect circadian metabolism might be an interesting therapeutic avenue. In this regard, compounds that act as agonists for REV-ERBs have been suggested to modulate the circadian clock and improve metabolic defects in mice [82, 83]. As our knowledge of NHR action in nutrient homeostasis continues to advance, these critical integrators of metabolism remain promising therapeutic targets for the treatment of metabolic disease.

## Abbreviations

BMAL1: Brain and muscle ARNT-like 1
CLOCK: Circadian locomotor output cycles kaput
CRY: Cryptochrome
ERRγ: Oestrogen-related receptor γ
FGF: Fibroblast growth factor
FXR: Farnesoid X receptor
LPL: Lipoprotein lipase
LXR: Liver X receptor
NHR: Nuclear hormone receptor
Nur77: Growth factor-inducible immediate early gene nur/77-like receptor
PGC1α: PPARγ co-activator 1α
PPAR: Peroxisome proliferator-activated receptor
REV-ERB: Reverse c-erbA
ROR: Retinoid orphan receptor
SCN: Suprachiasmatic nucleus
TZD: Thiazolidinedione
WAT: White adipose tissue
